# Distribution of genes related to Type 6 secretion system and lipooligosaccharide that induced ganglioside mimicry among *Campylobacter jejuni* isolated from human diarrhea in Thailand

**DOI:** 10.1186/s13099-020-00357-6

**Published:** 2020-04-09

**Authors:** Oralak Serichantalergs, Patcharawalai Wassanarungroj, Nuanpan Khemnu, Frédéric Poly, Patricia Guerry, Ladaporn Bodhidatta, John Crawford, Brett Swierczewski

**Affiliations:** 1 Department of Bacterial and Parasitic Diseases, Armed Forces Research Institute of Medical Sciences (AFRIMS), Bangkok, Thailand; 2 Enteric Diseases Department, Naval Medical Research Center, Silver Spring, MD USA; 3 US Army Medical Research Institute of Chemical Defense, Aberdeen, MD USA; 4 Bacterial Diseases Branch, Walter Reed Army Institute of Research, Silver Spring, MD USA

**Keywords:** *Campylobacter jejuni*, Capsule types, GBS, LOS, *hcp*, T6SS, Human diarrhea

## Abstract

**Background:**

*Campylobacter jejuni* (*C. jejuni*) is one of the most common bacteria responsible for human gastroenteritis worldwide. The mode of human transmission is foodborne infections due to consumption of contaminated food, especially poultry. Type 6 secretion systems (T6SS) were described recently as *Campylobacter* virulence mechanisms. Furthermore, infection sequelae associated with neurological disorders like Guillain–Barré (GBS) and Miller Fisher (MF) syndromes can become serious health problems in some patients after *Campylobacter* gastroenteritis. Our objective was to determine the distribution of these virulence genes among *C. jejuni* isolated from stool of human diarrhea.

**Methods:**

A total of 524 *C. jejuni* strains from travelers and pediatric cases of acute diarrhea in Thailand were selected for this study. All isolates belonged to one of 20 known capsule types and all were assayed by PCR for T6SS, a hemolysin co-regulated protein (*hcp*) gene, and GBS-associated genes (*cgtA*, *cgtB*, *cstII*_*HS19*_ and *cstII*_*HS2*_) which are involved in sialic acid production in the lipooligosaccharide (LOS) cores of *C. jejuni*. The distribution of these genes are summarized and discussed.

**Results:**

Of all isolates with these 20 capsule types identified, 328 (62.6%) were positive for *hcp*, ranging from 29.2 to 100% among 10 capsule types. The GBS-associated LOS genes were detected among 14 capsule type isolates with 24.4% and 23.3% of *C. jejuni* isolates possessed either *cstII*_HS19_ or all three genes (*cgtA*, *cgtB* and *cstII*_*HS19*_), which were classified as LOS classes A and B whereas 9.2% of *C. jejuni* isolates possessing *cstII*_*HS2*_ were classified as LOS class C. The *C. jejuni* isolates of LOS A, B, and C together accounted for 56.9% of the isolates among 14 different capsule types while 31.1% of all *C. jejuni* isolates did not possess any GBS-associated genes. No significant difference was detected from *C. jejuni* isolates possessing GBS-associated LOS genes among travelers and children, but changes between those with *hcp* were significant (p < 0.05).

**Conclusions:**

Our results suggested a high diversity of *hcp* and GBS-associated LOS genes among capsule types of *C. jejuni* isolated from Thailand.

## Introduction

*Campylobacter jejuni* is recognized as a major pathogen of gastroenteritis worldwide [1]. As illustrated by the latest epidemiological report, *Campylobacter* is the main cause of foodborne illness in Europe and the United States [1]. Campylobacteriosis cases range from 14 to 57 per 100,000 per year (US and Europe, respectively). In endemic regions, particularly in Southeast Asia, campylobacteriosis cases are estimated to be 10 times higher than in Europe and the USA. This high incidence of *Campylobacter* is well documented in children, travelers, and foreign military personnel from Thailand [2–4]. *C. jejuni* is considered a zoonotic disease. The major source of contamination is through consumption of improperly prepared or stored foods containing poultry, but other sources like unpasteurized milk or water have been documented. The infectious dose can be as low as 500 to 1000 bacteria [5]. Campylobacteriosis symptoms range from mild abdominal pain and mild to no diarrhea, to severe abdominal cramping, sometimes accompanied with fever, headache, myalgia, and large volumes of mucous and bloody diarrhea that can last for several days [6].

Despite a worldwide prevalence, there is a paucity of data regarding *C. jejuni* virulence factors. Nevertheless, flagella, cytolethal distending toxin (*cdt*), fibronectin binding protein (*cadF*), lipoprotein (*ceuE*) [7–9], and proteins involved in adherence and invasion acquired by plasmids (*pVir*) [10] contribute to virulence. The *Campylobacter* polysaccharide capsule (CPS) is the best characterized virulence factor. Mutants deficient in CPS production demonstrated lower adherence and binding in vitro, decreased serum resistance, and reduced colonization of ferret and chicken animal models [7, 11]. Phenotypic assays can be routinely applied for identification and classification of putative *C. jejuni* samples in laboratory settings [12]. Penner’s serotyping, developed in 1980s, is a well-known serotyping scheme for *C. jejuni* and *C. coli* that is based on capsular polysaccharide [13]. Molecular assays have replaced this method for routine *C. jejuni* and other *Campylobacter* spp. identification. Analyses of different CPS sequences were utilized to develop multiplex PCR assays to distinguish more capsule types among *C. jejuni* isolates [14–17]. This capsule typing scheme correlates well to Penner serotypes because the capsule structure is the major serodeterminant [14].

In addition to the virulence factors aforementioned, additional *C. jejuni* isolates were characterized and found to possess the recently described T6SS [18, 19]. The T6SS apparatus is composed of proteins which structurally and functionally are related to contractile components of bacteriophages [20]. The hemolysin co-regulated protein (Hcp) in *Pseudomonas aeruginosa* T6SS resembles the major component of the T4 phage tail base plate [21]. The T6SS contributes to bacterial pathogenesis in *Pseudomonas aeruginosa* [22], *Vibrio cholerae* [23], *Salmonella enterica* [24], *Helicobacter hepaticus* [25]*, Edwardsiella* spp. [26], *Burkoderia mallei* [27], as well as in *C. jejuni* [19]. *Campylobacter* T6SS plays an important roles in host cell adhesion, invasion, and persistent colonization in vivo [19]. T6SS was reported in *C. jejuni* isolated from clinical, poultry, and water sources, and its distribution varied in human and animal sources from different countries [28–30]. However, the distribution and role of T6SS pathogenesis in *C. jejuni* from human gastroenteritis has yet to be further determined.

A myriad of post infectious sequelae were linked to *C. jejuni* infections. These include autoimmune mediated Guillain–Barré (GBS) and Miller Fisher (MF) syndromes [31]. These autoimmune diseases are provoked by mimicry of *C. jejuni* lipooligosaccharides (LOS) structures that contain *N*-acetyl neuraminic (sialic acid), organized into structures that resemble human gangliosides [31]. Most *C. jejuni* strains contain genes for the endogenous synthesis of *N*-acetyl neuraminic acid, and there are specific genes involved in the biosynthesis of these ganglioside mimics [32]. The *cgtA* (β-1,4-*N*-acetylgalactosyl transferase) and *cgtB* (β-1,3-galactosyltransferase) and *cstII* (α-2,3 sialyltransferase) are required for molecular mimicry and are associated with human GBS. These genes were used as markers to screen for GBS-related strains of *C. jejuni* [33–35]. Multiple variants of *cstII* transferase were characterized in the *C. jejuni* LOS locus, including *cstII*_*HS19*_ and c*stII*_*HS2*_ [33]. The gene *cstII* has mono and bi-functional activities (α-2,3 and/or α-2,3/α-2,8-sialyltranferase), and both represent different specificity of enzymes involved in the transfer of the sialic acid residue to the LOS structure [32, 36]. Genes in LOS biosynthesis loci were studied from different origins of *C. jejuni*, and the LOS classes A, B, and C were classified for involvement in human ganglioside mimicry. Recently, LOS classes M and R were reported to possess genes similar to LOS classes A and D, including a gene encoding a sialyltransferase (*cstII*) [37].

The aim of this study was to determine the distribution of genes associated with T6SS and LOS implicated in GBS among different capsule types in clinical *C. jejuni* isolated from stool samples of travelers and children suffering diarrhea in Thailand.

## Materials and methods

### Clinical *C. jejuni* isolates and bacterial isolates

A total of 524 *C. jejuni* isolates were previously identified from stool samples collected from travelers (n = 313) and children (n = 211) suffering acute diarrhea in 1998–2003 and 2008–2010, respectively. *C. jejuni* isolated from traveler’s diarrhea studies were from traveling patients seeking care at Bumrungrad International Hospital, Bangkok in 2001–2002, and from military personnel participating in Cobra Gold Exercises during 1998–2003 in Thailand. *C. jejuni* isolated from stool cultures of children less than 5 years old with acute diarrhea from multiple regions in Thailand during 2008–2010, were included [17]. The studies used archived frozen *C. jejuni* isolates with appropriate consent for sample donation and future research use. All links or keys to subject name, number, and personal identifiable information were destroyed. All selected *C. jejuni* isolates were stored frozen at − 70 °C at AFRIMS.

All reference *C. jejuni* isolates used for PCR were provided by from the National Microbiology Laboratory, Public Health Agency of Canada, Winnipeg, Manitoba, Canada. These include ATCC-43429, -43432, -43434, -43438, -43439, -43456, and LIO 87 for LOS class differentiation. The *C. jejuni* strains ATCC -43431, BH-0142, NTCT-11168, and 81-176 were reference strains for T6SS previously identified from collection strains at AFRIMS.

### Growth and genomic DNA purification of *C. jejuni* isolates

*Campylobacter jejuni* were subcultured on sheep blood agar plates, using aliquots of the frozen isolates. The plates were incubated under microaerobic conditions (85% N_2_, 10% CO_2_, and 5% O_2_) at 42 °C overnight. Genomic DNA extraction from *C. jejuni* isolates were performed using DNeasy tissue extraction kits (Qiagen, MD, USA). The quantity and quality of DNA was estimated using a NanoDrop (Thermo scientific, MA, USA) and agarose gel electrophoresis. Gels images were visualized using ethidium bromide and a gel documentation system (Syngene, Cambridge, UK).

### PCR for *C. jejuni* capsule types

Primer sets specific for *C. jejuni* capsule types were used according to previously published articles [16, 17]. In brief, four primer sets (35 capsule primer pairs) recognized a total of 47 Penner serotypes and complex types. All 524 *C. jejuni* genomic DNA were analyzed with four multiplex PCR sets (Alpha, Beta, Gamma, and Delta). Multiplex PCR and step cycles followed previously reported standardized procedures [16, 17]. Primer pairs for the 331 bp *lpxA* gene (involved in lipid A biosynthesis) were used as an internal control for *C. jejuni*. All PCR amplification products were evaluated using a 2.0% agarose gel (Thermo Fisher Scientific -Invitrogen, MA, USA) in 0.5× Tris–Borate EDTA buffer at 150 volts for 60 min. The DNA bands were visualized and photographed under UV light after the gel was stained with ethidium bromide. The amplification products were interpreted accordingly as described in [16].

### PCR detection for T6SS gene

An *hcp* sequence from *C. jejuni* T6SS was obtained from GenBank, accession number JX436640. Primers specific to the *hcp* gene were designed in this study using Primer3 software [38]. The *hcp* forward (hcp_1) and reverse (hcp_2) primer sequences are 5′-CAAATGCGCAAGAGTCAAGT-3′ and 5′-TAAGCTTTGCCCTCTCTCCA-3′, respectively. Primers specific for *lpxA* was included in these PCR as an internal control. The DNA extracts of *C. jejuni* ATCC 43431 and clinical isolates BH-01-142 were used as positive controls for *hcp* [19]. The negative control strains were *C. jejuni* NCTC 11168 and 81-176 [18, 28]. Multiplex PCR was performed on a Nexus GX2 thermal cycler (Eppendorf, NY, USA) using AmpliTaq Gold DNA Polymerase (Thermo Fisher Scientific, MA, USA). The PCR step cycles for *hcp* were 1 cycle for 94 °C for 5 min, 28 cycles for 94 °C for 1 min, 52 °C 1 min, 72 °C for 1 min, and a final cycle at 72 °C for 10 min. A 100 bp DNA ladder (Thermo Fisher Scientific, MA, USA) was applied as a molecular marker. All PCR amplification products were evaluated on a 2.0% agarose gel (Invitrogen) at 150 volts for 60 min. The DNA bands were visualized and photographed under UV light after the gel was stained with ethidium bromide. The estimated size of the *hcp* amplified products was 133 bp.

### PCR detection for GBS-associated LOS genes

Four primer pairs specific for the LOS genes implicated in ganglioside mimicry: *cgtA* (β-1,4-*N*-acetylgalactosyltransferase), *cgtB* (β-1,3-galactosyltransferase), *cstII* (α-2,3 sialyltransferase, and/or *cstII* (α-2,3/α-2,8 sialyltransferase), were used, as previously described [35]. Multiplex PCR was performed on a thermal cycler using AmpliTaq Gold DNA Polymerase. The PCR step cycles were: 94 °C for 5 min, 94 °C for 1 min, 53 °C (*cgtA* and *cgtB*, *cstII*_*HS19*_ and *cstII*_*HS2*_) for 1 min, and 72 °C for 1 min for 28 cycles, with a final 10 min extension at 72 °C. Positive controls for the GBS-associated LOS genes in PCR were genomic DNA from *C. jejuni* ATCC 43432 (LOS-A1), ATCC 43438 (LOS-A2), ATCC 43456 (LOS-B1), ATCC 43439 (LOS-B2), NCTC 11168, ATCC 43429 (LOS-C), LIO 87 (LOS-D), and ATCC 43434 (LOS-E) to determine LOS classes A-E in *C. jejuni* as previously described [32, 39]. The internal PCR control was *C. jejuni* specific *lpxA.* All PCR amplification products were applied and evaluated on a 3.0% agarose-1000 gel (Invitrogen) at 150 volts for 100 min. A 100 bp DNA ladder was used as a molecular marker. The DNA bands were visualized and photographed under UV light after the gel was stained with ethidium bromide. The sizes of amplified products from *cgtA*, *cgtB cstII*_*HS19*_, and *cstII*_*HS2*_ were 527, 502, 400, and 417 bp, respectively. Primers of the four genes, and one internal control gene herein distinguished control isolates as follows: ATCC 43432 and ATCC 43456, representing LOS classes A1 and B1, were positive for all three genes c*gtA*, *cgtB*, and *cstII*_*HS19*_ whereas the control strains ATCC 43438 and ATCC 43449 representing LOS classes A2 and B2 were only positive for gene *cstII*_*HS19*_. Two control strains, NCTC 11168 and ATCC 43429 both representing LOS class C, were positive for only *cstII*_*HS2*_. Two control strains LIO 87 and ATCC 43434 representing LOS classes D and E, respectively were negative for all four genes studied. Thus, *C. jejuni* isolates possessing *cstII*_*HS19*_ or all three genes (c*gtA*, *cgtB*, and *cstII*_*HS19*_) were characterized as either LOS class A or class B, whereas isolates positive for only *cstII*_*HS2*_ were classified as LOS class C. Isolates negative for all genes were classified as non-LOS classes A, B, or C.

### Sequencing of PCR amplified products and analysis

Amplification products of selected clinical *C. jejuni* isolates positive for *hcp, cgtA, cgtB*, *cstII*_*HS19*_, and *cstII*_*HS2*_ were purified for sequencing using the Wizard SV gel and PCR clean-up system (Promega, WI, USA). After purification, the amplified products for *hcp* and the four GBS-associated LOS genes were submitted for sequencing (Macrogen, South Korea). The amplified products of *hcp*, *cgtA*, *cgtB*, *cstII*_*HS19*_, and *cstII*_*HS2*_ were edited and assembled by Sequencher version 5.0 (Gene Codes Corporation, MI, USA). The nucleotide sequences were analyzed with BLAST (http://www.ncbi.nlm.nih.gov/BLAST).

### The association of *C. jejuni* capsule types, *hcp*, and GBS-associated LOS genes

The distribution of *hcp* and GBS-associated LOS genes detected in *C. jejuni* isolated from travelers and child diarrhea cases were tested for statistical differences using Fishers exact test from GraphPad software [40]. A *p* value < 0.05 was considered statistically significant.

## Results

### Clinical *C. jejuni* isolates and capsule type distribution

**Fig. 1:**
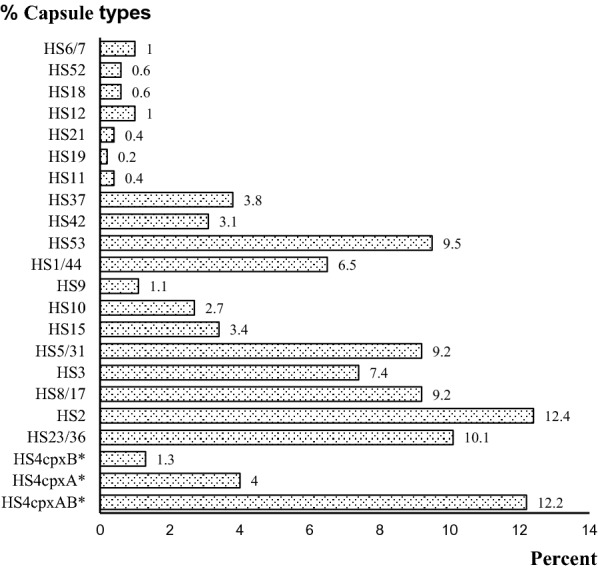
Distribution of capsule types in 524 *C. jejuni* isolated from Thailand. *HS4cpxA, HS4cpxB, and HS4cpxAB are HS4cpx subtypes

A total of 524 archived *C. jejuni* isolates were previously characterized for capsule typing [16], and these comprised 20 capsule types. More than 82% (n = 429) of all isolates belonged to eight major capsule types: HS4, HS2, HS23/36, HS53, HS5/31, HS8/17, HS3, and HS1/44, as indicated in Fig. 1. The remaining 95 isolates, ranging from 1 to 3.8% were capsule types HS37, HS15, HS42, HS10, HS9, HS6/7, and HS12; and < 1% for capsule types HS52, HS18, HS21, HS11, and HS19, each. Additionally, 92 *C. jejuni*-HS4 isolates gave the following HS4 subtypes: HS4_*cpxAB*_ (n = 64, 12.2%); HS4_*cpxA*_ (n = 21, 4.0%), and HS4_*cpxB*_ (n = 7, 1.3%).

Different proportions and significance of capsule types among *C. jejuni* isolates from travelers and children were observed in these two populations (Table 1). The prevalence of capsule types in these isolates more frequent in travelers compared to children: HS4_*cpxAB*_, HS23/36, HS53, and HS15. In contrast, *C. jejuni* HS2, HS8/17, HS1/44, and HS9 showed greater prevalence in children compared to travelers.

**Table 1:** Distribution of 524 *C. jejuni* isolates and capsule types from two studies groups

Capsule types	Travelers (n = 313)	%	Children (n = 211)	%	*p*-value^b^
HS4_*cpx*_^a^	63	20.1	29	13.7	NS
HS4_*cpxAB*_	47	15.0	17	8.1	0.0203
HS4_*cpxA*_	9	2.9	12	5.7	NS
HS4_*cpxB*_	7	2.2	0	0	N/A
HS2	23	7.3	42	19.9	0.0001
HS23/36	48	15.3	5	2.4	0.0001
HS53	43	13.7	7	3.3	0.0001
HS5/31	23	7.3	25	11.8	NS
HS8/17	18	5.8	30	14.2	0.0017
HS3	18	5.8	21	10	NS
HS1/44	14	4.5	20	9.5	0.0292
HS37	13	4.2	7	3.3	NS
HS15	16	5.1	2	0.9	0.0127
HS42	16	5.1	0	0	N/A
HS10	7	2.2	7	3.3	NS
HS9	1	0.3	5	2.4	0.0413
HS6/7	0	0	5	2.4	N/A
HS12	2	0.6	3	1.4	NS
HS52	3	1	0	0	N/A
HS18	1	0.3	2	0.9	NS
HS21	1	0.3	1	0.5	NS
HS11	2	0.6	0	0	N/A
HS19	1	0.3	0	0	N/A
Total	313	100.0	211	100.0	

*NS* not significance, *N/A* not applicable

^a^HS4_*cpx*_ composed of HS4_*cpxAB*_, HS4_*cpxA*_, and HS4_*cpxAB*_

^b^Fisher exacts test

### Distribution of the *hcp* gene

**Fig. 2:**
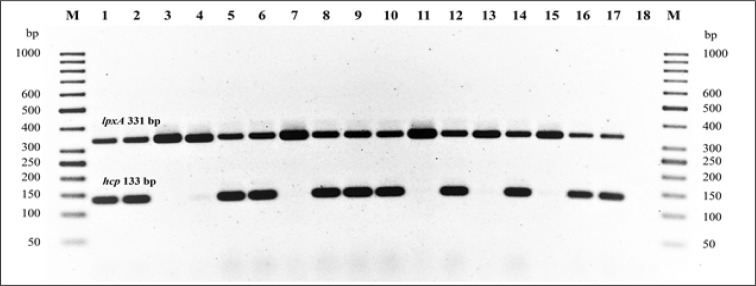
Gel electrophoresis of amplified products of *hcp* (133 bp) and *lpxA* (331 bp). Lane M: 100 bp DNA maker; Lane 1, 2: positive controls ATCC43431, BH-01-142; Lane 3, 4: negative controls NCTC 11168 and 81-176; Lane 5–11: clinical *C. jejuni* isolated from travelers; BH-01-270 (HS3, *hcp* positive), BH-01-39 (HS2, *hcp* positive), BH-01-6 (HS19, *hcp* negative), CG-02-3018, (HS4_*cpxAB*_, *hcp* positive), CG-3-3030 (HS 23/36, *hcp* positive), CG-03-4020 (HS23/36, *hcp* positive) and CG-98-U-22 (HS3, *hcp* negative); Lane 12–17: clinical *C. jejuni* isolated from children; CR-08-181 (HS2, *hcp* positive), KR-08-132 (HS53, *hcp* negative), PN-08-207 (HS2, *hcp* positive), PN-08-083 (HS4, *hcp* positive), PN-08-128 (HS23/36, *hcp* positive), and PN-08-230 (HS3, *hcp* positive); Lane 18: negative control (nuclease-free water)

**Fig. 3:**
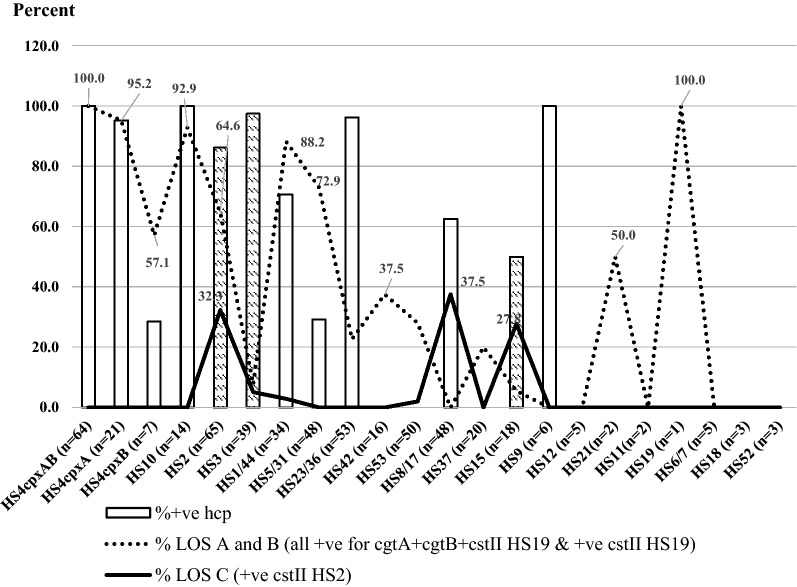
Distribution of *hcp* and GBS-associated LOS genes amongst *C. jejuni* capsule types. A bar graph and two line graphs represent the percent distribution of *hcp* and LOS (class A or B) and class C genes, respectively among numbers of *C. jejuni* isolates of each capsule type. A bar graph with a filled pattern show *C. jejuni* capsule types associated both *hcp*, LOS classes A, B, and C

Figure 2 represents the amplified 133-bp PCR products of the *hcp* gene detected in 13 selected *C. jejuni* isolated from different capsule types in travelers and children. As shown in Table 2, the *hcp* gene was detected in 62.6% (328/524) of all *C. jejuni* isolates but less frequently in travelers than children 55.6% (174/313) versus 73.0% (154/211), respectively (*p *= 0.0001). Only *C. jejuni* HS8/17-*hcp* positive isolates were detected more often in children (86.7%; 26/30) than in travelers (22.2%; 4/18), while we did not observe any *hcp* significance in other capsule type isolates from travelers and children. The prevalence of *hcp* detected varied from 28.6 to 100% in isolates of 10 capsule types: HS4*cpx* (all subtypes), HS23/36, HS2, HS8/17, HS3, HS5/31, HS1/44, HS15, HS10, and HS9, and no *hcp* genes were detected from the remaining 10 *C. jejuni* capsule types: HS53, HS42, HS37, HS11, HS12, HS18, HS19, HS21, HS52, and HS6/7 (Fig. 3).

**Table 2:** Distribution of *C. jejuni*, *hcp*, and GBS-associated LOS genes in travelers and children

*C. jejuni* traits	LOS	Total (n)	%	Travelers (n)	%	Children (n)	%	*p*-value^a^
*C. jejuni*, *hcp*	–	328 (524)	62.6	174 (313)	55.6	154 (211)	73.0	0.0001
HS8/17, *hcp*	–	30 (48)	62.5	4 (18)	22.2	26 (30)	86.7	0.0001
*C. jejuni, cgtA*, *cgtB*, *cstII*_*HS19*_	A, B	122 (524)	23.3	79 (313)	25.2	43 (211)	20.4	NS
*C. jejuni, cstII*_*HS19*_	A, B	128 (524)	24.4	71 (313)	22.7	51 (211)	24.2	NS
*C. jejuni, cstII*_*HS2*_	C	48 (524)	9.2	19 (313)	13.1	29 (211)	13.7	NS
*C. jejuni, cgtB*	N/A	6 (524)	1.3	6 (313)	1.9	0 (211)	0	N/A
*C. jejuni, cgtA, cgtB*	N/A	1 (524)	0.2	0 (313)	0	1 (211)	0.5	N/A
*C. jejuni, cgtB*, *cstII*_*HS19*_	N/A	34 (524)	6.5	32 (313)	10.2	2 (211)	1.0	0.0001
*C. jejuni, cstII*_*HS19*_, *cstII*_*HS2*_	N/A	6 (524)	1.1	6 (313)	1.9	0 (211)	0	N/A
*C. jejuni, cgtB*, *cstII*_*HS19*_, *cstII*_*HS2*_	N/A	11 (524)	2.1	11 (313)	3.5	0 (211)	0	N/A
*C. jejuni, cgtA*, *cgtB*, *cstII*_*HS19*_*, cstII*_*HS2*_	N/A	5 (524)	0.9	5 (313)	1.6	0 (211)	0	N/A

*NS* not significance, *N/A* not applicable

^a^Fisher exact test was calculated for significance, comparing travelers and children

### Distribution of GBS associated-LOS genes

**Fig. 4:**
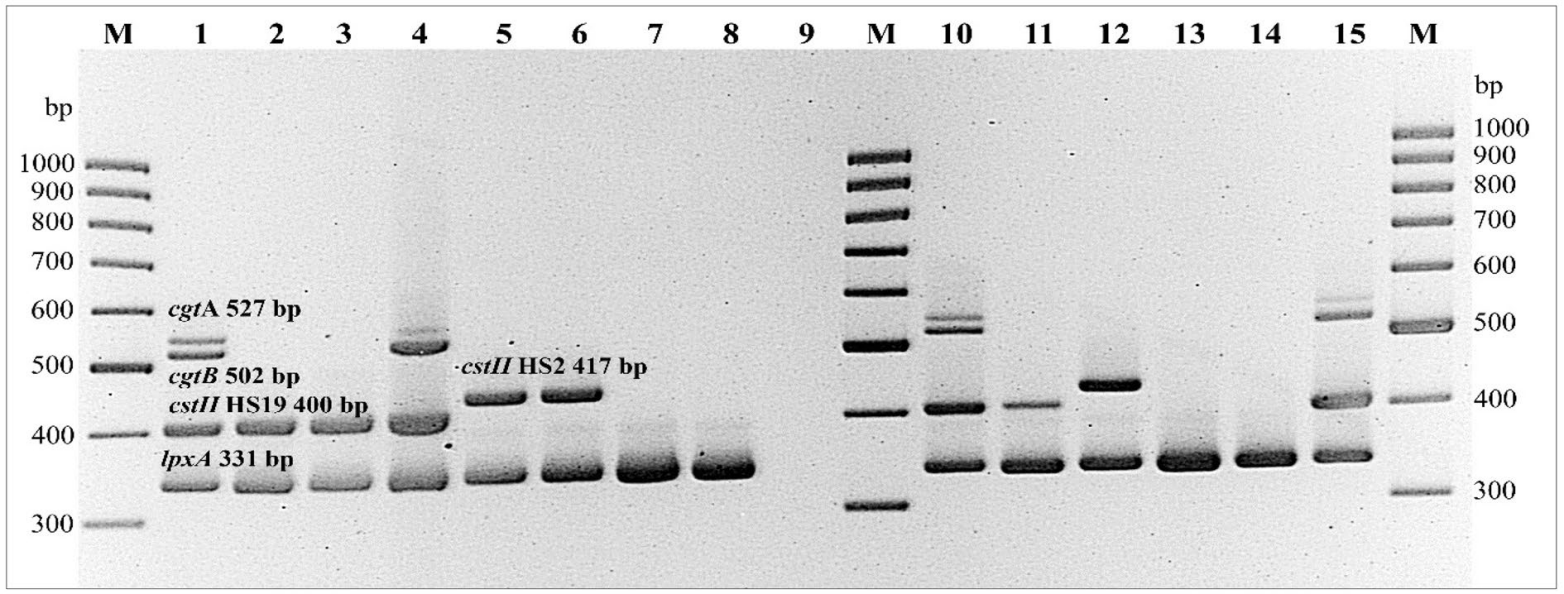
Gel electrophoresis of amplified products of GBS-associated LOS genes from selected *C. jejuni* isolates from travelers and children. PCR products representing *cgtA* (527 bp), *cgtB* (502 bp), *cstII*_*HS2*_ (417 bp), *cstII*_*HS19*_ (400 bp), and *lpxA* genes from *C. jejuni* isolates in this study. Lane M, 100 bp DNA maker; Lane 1, ATCC 43432 (LOS class A1); Lane 2, ATCC 43438 (LOS class A2); Lane 3, ATCC 43449 (LOS class B2); Lane 4, ATCC 43456 (LOS class B1); Lane 5, 6: NCTC 11168 and ATCC 43429 (LOS class C); Lane 7, LIO87 (LOS class D); Lane 8, ATCC 43434 (LOS class E); Lane 9, negative control (nuclease-free water); Lane 10, 11, and 13, amplified product of selected *C. jejuni* isolates from travelers CG-01-09510 (HS2), CG-03-4049 (HS23/36), and CG-99-8135 (HS23/36), respectively; Lane 12, 14, and 15, amplified products of selected *C. jejuni* isolates from children, SR-08-150 (HS2), BH-01-193 (HS15), and KR-08-207 (HS4_*cpxAB*_), respectively

Percent distribution of four GBS-associated LOS genes are described in Table 2. There were no significant differences between a given gene, or group of genes, among travelers compared to children, except for *C. jejuni* isolates that harbored *cgtB* and *cstII*_*HS19*_ (*p *< 0.0001)*. C. jejuni* isolates that harbored *cstII*_*HS19*_ (24.4%) alone, or three genes for *cgtA*, *cgtB* and *cstII*_*HS19*_ (23.3%), were the two most common patterns in this study. Six variants of 29 *C. jejuni* isolates possessed the following gene variations: (*cgtA, cgtB*), (*cgtA*, *cgtB*, *cstII*_*HS19*_, and *cstII*_*HS2*_), and (*cstII*_*HS19*_, *cstII*_*HS2*_). The *cgtB* and (*cgtA*, *cgtB*, *cstII*_*HS19*_*, cstII*_*HS2*_) groups were observed at a very low frequency from 0.2 to 2.1%, respectively. In addition, *C. jejuni* isolates harboring *cstII*_*HS2*_ and *cgtB *+ *cstII*_*HS19*_ were detected at 9.2% and 6.5%, respectively. Overall, 163 of 524 (31.1%) of *C. jejuni* isolates lacked all four GBS-associated LOS genes, and 78 and 85 were isolates obtained from travelers and children, respectively (Fig. 3). All 163 *C. jejuni* isolates belonged to 18 different capsule types: HS1/44, HS11, HS12, HS18, HS15, HS21, HS23/36, HS31, HS37, HS4*cpxA*, HS4*cpxB*, HS 42, HS52, HS53, HS6/7, HS8/17, and HS9, whereas all five capsule types isolates: HS9, HS 12, HS6/7, HS18 and HS52 always lacked all four GBS-associated LOS genes in this study. Figure 4 depicts amplified PCR products corresponding to *cgtA, cgtB*, *cstII*_*HS19*_, and *cstII*_*HS2*_ from selected *C. jejuni* isolated from travelers and children in this study.

### Association of capsule types of *C. jejuni* isolates with *hcp* and GBS-associated LOS genes

Figure 3 depicts a comparison of *hcp*, GBS-associated LOS genes and each of *C. jejuni* capsule type in this study: *hcp*, (c*gtA*, *cgtB* and *cstII*_*HS19*_), *cstII*_*HS19*_, and *cstII*_*HS2*_. A bar graph displays the percent of *hcp*, and two line graphs represent percent of GBS-associated LOS genes: [(*cgtA*, *cgtB* and *cstII*_*HS19*_), *cstII*_*HS19*_] and *cstII*_*HS2*_.

A high percentage (60–100%) of *C. jejuni* isolates in capsule types HS4_*cpxAB*_, HS4_*cpxA*_, HS4_*cpxB*_, HS10, HS2, and HS1/44 were associated with *hcp* and GBS-associated classes A and B LOS genes. In contrast, *C. jejuni* HS3, HS23/36 and HS15 isolates possessed a lower percentage of LOS class A and B as compared to *hcp*. *C. jejuni* HS8/17 and HS9 isolates harbored only *hcp* but did not have LOS classes A and B. *C. jejuni* HS42, HS53, HS37, HS21, and HS19 isolates had GBS-associated LOS genes represented LOS class A and B, but not *hcp* (dot line versus bar graph displays).

In the line graph display, three *C. jejuni* isolates capsule types: HS2, HS8/17, and HS15 possessed only *cstII*_*HS2*_, representing LOS class C as 32.3%, 37.5%, and 27.8%, respectively. These *C. jejuni* isolates from the three capsule types also harbored *hcp*, whereas *C. jejuni* isolates from HS2, HS3, HS1/44, HS53, and HS15 possessed both LOS classes A, B, or C.

## Discussion

In this study, HS4 and HS2 were the most common *C. jejuni* capsule types from traveler and pediatric diarrhea cases, respectively. These two capsule types were the two most common Penner serotypes in global human *C. jejuni* isolates, confirming previous reports [41]. Other capsule types exhibited varied distribution among isolates from travelers and children. The study of traveler’s diarrhea isolates included *C. jejuni*, of which > 80% were obtained from stool samples of military personnel attending annual exercises from different locations in Thailand. A point-source of infection of an identical *C. jejuni* clone isolated from the same type of food consumed might attribute a distribution of one capsule type over the others (e.g., HS53, HS23/36). The different capsule types seen in travelers may be affected by temporal variations during the exercises of military personnel, and by the contaminated food consumed by travelers at the visiting sites (e.g., resort or hotel), but this was not seen in isolates from children in which samples were collected over a 2 year period. Although *C. jejuni* isolates from these two studies were from more than 5 years apart, the five most common capsule types from traveler isolates (HS4, HS2, HS5/31, HS8/17, and HS3) and Thai children isolates (HS2, HS 8/17, HS4, HS5/31, and HS3) were comparable to capsule serotypes of global *C. jejuni* isolates reported previously [41].

T6SS are bacteriophage-like structures which secrete effector molecules into eukaryotic cells or other bacteria [20, 42]. The presence of a secreted effector, the *hcp* needle protein, in culture supernatants suggested contribution of this protein to virulence of *C. jejuni* strains [18, 19]. The *hcp* gene was used as a surrogate for the presence of T6SS in *C. jejuni*. Using this screening method on different *C. jejuni* isolates, heterogeneous results were reported. Harrison et al. [28] demonstrated the prevalence of *hcp* in 60.6% and 33.3% from clinical *C. jejuni* isolates in Vietnam and Thailand, respectively. However, only three human *C. jejuni* isolates from Thailand were studied. None of the T6SS genes were detected in 34 *C. jejuni* isolated from human diarrheal cases in Pakistan [29]. In our study, *hcp* was found in 62.6% of the 524 cases, and this proportion was similar to studied isolates in Vietnam (60.6%) and Egypt (57.6%) [28, 43].

The *hcp* gene was associated with 10 capsule types of *C. jejuni* isolates whereas a variable *hcp* prevalence was reported by others. Sainato et al. [43] reported a *hcp* gene prevalence of 33.3% in HS2 isolates, but up to 86.2% was noted in this study. Moreover, numbers of *hcp*-*C. jejuni* isolates were more significant in children than travelers (22.2%, 86.7%; *p *= 0.0001), especially in HS8/17 isolates. Different distributions of the *hcp* gene were also observed among HS4*cpx* isolates (28.5% of HS4_*cpxB*_, 92.5% HS4_*cpxA*_, and 100% of HS4_*cpxAB*_). Our study was limited in that we tested for only one gene marker in T6SS, while the T6SS locus composed of at least 13 ORFs [18]. A fully-functional T6SS was only reported in two *C. jejuni* strains in published articles [18, 19]. Further investigation on more conserved genes in the T6SS locus and expression of their encoded proteins may implicate the contribution of T6SS to *Campylobacter* virulence.

*Hcp* was previously reported to be associated with bloody diarrhea [28]. We found no significant association between *hcp* and *C. jejuni* isolates of any specific capsule type. This is similar to the finding by Agnetti et al. [44], where no significant difference in the clinical manifestations and the course of disease between patient with hcp-positive and -negative *C. jejuni* isolates. Similarly, the presence of *hcp* gene in *C. jejuni* and *C. coli* from stool did not have any significant association to clinical outcome in Egyptian children [43].

*Campylobacter jejuni* and their LOS classes could be distinguished by different LOS biosynthesis locus, as referenced by others [32, 33, 39]. Parker et al, reported *orf* locus 7ab and 7c to encode for *cstII* and *cstIII*, respectively. In our study, the primers for four genes, c*gtA*, *cgtB*, *cstII*_*HS19*_ and *cstII*_*HS2*_, were used following Nachamkin, et al. [35]. The amplified products representing clinical *C. jejuni* of different capsule types, shown in Fig. 4, were sequenced and analyzed with BLASTn to identify each gene. The alignment matched within locus of *cgtA*, *cgtB*, *cstII, and cstIII,* respectively. In this study, we focused on LOS classes A to C that can cause sialylated LOS synthesis resulting in ganglioside mimicry, which are crucial for ganglioside mimicry from various *C. jejuni* strains, as previous reported [32, 39, 45–47]. The GBS genes for LOS classes A, B are predominately amongst *C. jejuni* HS19 isolates [47]. All 524 *C. jejuni* isolates were associated with gastroenteritis (GI). C*stII*_*HS19*_ was previously reported in GBS-HS19 isolates more than in GBS-non HS19 isolates [35]. In this study, almost 50% (23.3 and 24.4% in Table 2) of the GI-isolates were classified as LOS classes A or B, but only one isolate was HS19 serotype. GBS was commonly associated with HS1/44-, HS2-, and HS4-*C*. *jejuni* antecedent infections in the Netherlands, whereas HS19-, HS 23/36-, and HS41-*C. jejuni* were associated with GBS in Bangladesh, Japan, and South Africa [47]. Our study demonstrated a high prevalence of HS1/44, HS2, HS23/36, and HS4_*cpx*_, but the HS41 serotype was not detected from the GI-isolates. Distribution of LOS classes A, B, and C in *C. jejuni* has been previously demonstrated, as 17%, 32%, and 19%, respectively, but there are data linked to only 5 Penner serotypes from these *C. jejuni* isolates [47]. Parker et al. [39] reported that 64% of non-GBS associated isolates were in LOS classes (A, B, or C), and this is comparable to our study that 56.9% of *C. jejuni* isolated were in LOS classes A, B, and C. A limitation of our study was that the primers used for LOS associated GBS genes could not amplify gene targets to differentiate LOS subclasses A1, A2, B1 and B2, and other LOS loci diversity [37, 39, 48, 49]. More primers specific to other LOS classes should be included to characterize these *C. jejuni* isolates in more depth, especially in 63 *C. jejuni* isolates with unclassified LOS locus.

Differences in association of CPS and MLST types were also seen among these *C. jejuni* strains compared to published studies. Heikema et al. [47] observed that HS23/36 isolates from GBS patients were associated with a MLST clonal complex (CC)-403, -42, and -206, but most of HS23/36-GI isolates in this study were MLST CC-52 (> 90%). Similarly most of GBS-HS2 isolates in the Heikema study were MLST CC-21, but our GI-HS2 isolates were MLST-CC-464 (83%) and unassigned CC (27%). HS4-GBS and enteritis isolates were MLST CC-48, -61, -206, and -508 (Heikema), while most of GI-isolates in our study were MLST-52, -460, and unassigned CC (data not shown). This suggested there was a selective and divergent clone lineage of *C. jejuni* GBS- and GI-strains; however, many other factors likely also contribute to *Campylobacter* virulence.

## Conclusion

This data provides information on the diversity of *hcp* and LOS genes implicated with GBS-associated *C. jejuni* capsule types in travelers and children, as compared to other studies. A high prevalence of T6SS and GBS-associated LOS genes was found in certain *C. jejuni* capsule types: HS4_*cpxAB*_, HS4_*cpxA*_, HS2, HS1/44, and HS10, which may suggest an increase fitness of these *Campylobacter* since most isolates contained *hcp* and LOS classes A, B, or C genes, which are crucial for survival in the host and promote the synthesis of sialylated LOS. However, variations in clonal strains, different biological LOS functions, as well as host and environment factors, may be involved in triggering an autoimmune response and need further study. The understanding of global capsule types, antigenic epitopes, and virulence determinants may contribute towards future development of targeted therapeutics against *C. jejuni* diarrhea.

## Data Availability

All the supporting the findings are presented in the manuscript.

## Abbreviations

T6SS: Type 6 secretion system
GBS: Guillain–Barré syndrome
MF: Miller Fisher
LOS: Lipooligosaccharide
MLST: Multilocus sequence typing
CC: Clonal complex
GI: Gastroenteritis
CPS: Capsule polysaccharide
ORF: Open reading frame
